# miR-33a and Its Association with Lipid Profile in Patients with Carotid Atherosclerosis

**DOI:** 10.3390/ijms24076376

**Published:** 2023-03-28

**Authors:** Marine M. Tanashyan, Alla A. Shabalina, Polina I. Kuznetsova, Anton A. Raskurazhev

**Affiliations:** Research Center of Neurology, 80, Volokolamskoe Shosse, 125367 Moscow, Russia

**Keywords:** carotid atherosclerosis, microRNA, cholesterol, low-density lipoprotein

## Abstract

Atherosclerosis is a chronic inflammatory disease with a complex, multifactorial pathogenesis, which includes lipid metabolism alterations. miR-33a is a microRNA that plays a key role in cholesterol efflux and promotes atherosclerosis, yet its relationship with lipid markers in carotid atherosclerosis (CA) remains unclear. The objective is to evaluate possible associations between miR-33a expression and lipid biomarkers in patients with CA. This was a prospective study that included 61 patients (median age 66.0 years, 55.7% male) with evidence of CA. Lipid profile (total cholesterol, triglycerides [TG], high-density lipoprotein [HDL] and low-density lipoprotein [LDL] cholesterol) was analyzed. Extraction and quantification of miR-33a-5p/3p was performed according to protocol. Patients were further divided depending on the target LDL level (<1.8 mmol/L). Patients with CA had relatively favorable LDL levels with a median of 2.0 mmol/L. Both miR-33a-5p and miR-33a-3p levels were lower in patients with less than targeted LDL levels (37.4 and 38.3 vs. 41.8 and 42.5 respectively, *p* < 0.05). A significant positive correlation between expression levels of miR-33a-5p/3p and degree of carotid stenosis was found (r = 0.44 and r = 0.38 respectively, *p* < 0.05). In a univariate linear regression model miR-33a-3p/5p was positively associated with LDL cholesterol (*p* = 0.02). miR-33a up-regulation is associated with CA and may, in fact, be a key player by targeting cholesterol metabolism. A decrease in LDL cholesterol (<1.8 mmol/L) corresponded to lower levels of miR-33a, yet the direction and causality of this association remains unclear.

## 1. Introduction

Atherosclerosis is a chronic inflammatory disease with a complex, multifactorial pathogenesis. Initiation of atherosclerosis relies heavily on the subintimal (subendothelial) accumulation of low-density lipoprotein (LDL) cholesterol which then becomes oxidized and promotes further pathological alterations [1]. MicroRNAs (miRs) are a novel class of biomarkers that are substantially involved in atherosclerosis progression, and may present valuable diagnostic and therapeutic modalities. Lipid metabolism is one of the key components of atherogenesis, and it was previously demonstrated that a family of microRNAs—namely, miR-33a/b—play a pivotal role in cholesterol efflux targeting the ATP-binding cassette transporter A1 (ABCA1) [2]. In vivo studies have shown that antisense inhibition of miR-33a results in atherosclerotic plaque regression in mice by promoting reverse cholesterol transport and increasing high-density lipoprotein (HDL) levels [3].

Carotid atherosclerosis (CA) remains a major risk factor for ischemic stroke, and the prevalence of carotid stenosis in people aged 30–79 years is 1.5%, which approximates to 58 million [4]. A number of biomarkers have been described in the last few decades which reflect various pathological processes underlying CA progression, yet few have been implemented into real-world clinical practice. We previously described the expression pattern of selected miRs in a cohort of CA patients with varying degrees of carotid stenosis, identifying a highly specific and sensitive marker of advanced CA—miR-126-5p [5]. In this article, we wanted to focus more thoroughly on the interactions of miR-33a expression and lipid biomarkers in CA patients.

## 2. Results 

The main demographic characteristics are presented in Table 1. We further divided our study cohort depending on achieving target LDL levels (<1.8 mmol/L). 

Patients with less than target LDL levels had lower BMI (26.4 vs. 27.7 kg/m^2^, *p* = 0.03) and had a more pronounced history of cardiac disease (i.e., myocardial infarction [30.8% vs. 5.7%, *p* = 0.02] and atrial fibrillation [23.1% vs. 2.9%, *p* = 0.04]). Incidentally, the rates of prior stroke did not differ statistically significant between groups (38.5% vs. 28.6%, *p* = 0.59).

The lipid profile is shown in Table 2. Overall, patients with CA had relatively favorable LDL levels with a median of 2.0 mmol/L. The statistically significant difference between total cholesterol levels occurred probably due to the LDL-based group division.

**Figure 1:**
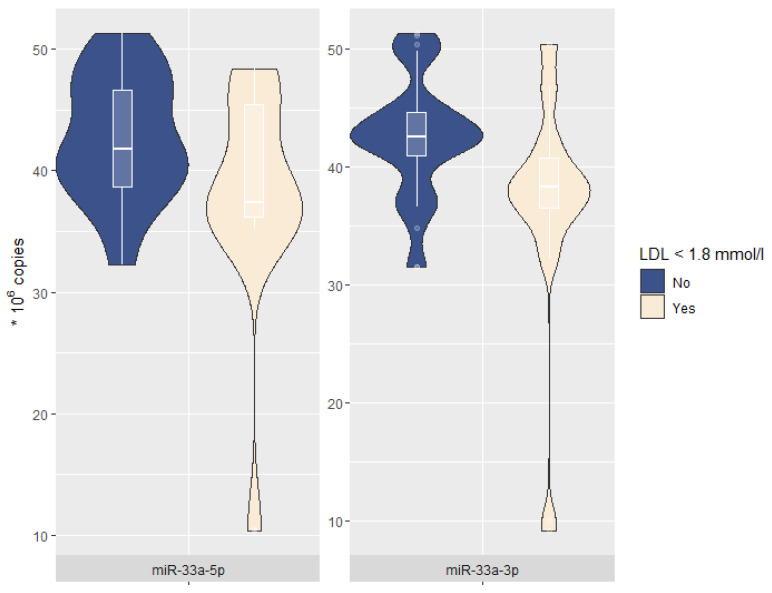
miR-33a-5p/3p expression levels according to target LDL levels. Combined violin-and-box-plot: the horizontal lines corresponds to the median (**center**) and first and third quartiles (**lower** and **upper**, respectively); the shape of the violin plot reflects the distribution of the variable.

miR-33a expression levels are presented in Table 3 and Figure 1. Both miR-33a-5p and miR-33a-3p levels were lower in patients with less than targeted LDL levels (37.4 and 38.3 vs. 41.8 and 42.5, respectively, *p* < 0.05).

**Figure 2:**
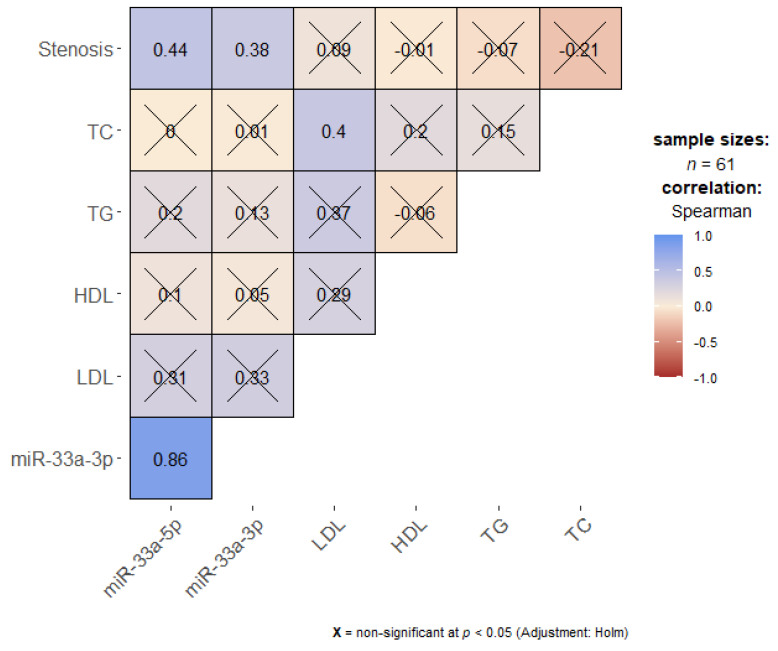
Correlation plot (non-parametric, the number in each box is the Spearman coefficient). Crossed-out are unsignificant (at *p* < 0.05) correlations. LDL—low-density lipoprotein, HDL—high-density lipoprotein, TC—total cholesterol, TG—triglycerides. The color of the box corresponds to the strength of the correlation (refer to the color legend).

In order to establish if miR-33a-5p/3p expression levels could be associated with lipid biomarkers an exploratory correlation analysis was carried out (Figure 2).

A significant positive correlation between expression levels of miR-33a-5p/3p and degree of carotid stenosis was found (r = 0.44 and r = 0.38, respectively, *p* < 0.05). Total cholesterol was also positively correlated with LDL levels (r = 0.4, *p* < 0.05).

A univariable linear regression analysis was then performed with miR-33a-5p/3p levels as independent variables in order to establish their predictive value (Table 4).

miR-33a-5p/3p expression is positively associated with LDL levels in our study cohort (*p* = 0.02). 

## 3. Discussion

High level of LDL cholesterol is considered one of the most significant risk factors for cerebrovascular disease: it is ranked third highest globally by the number of disability-adjusted life-years due to ischemic stroke [6]. Aggressive LDL-lowering treatment has been demonstrated to obtain clinical benefit in certain high-risk cohorts—e.g., in a secondary prevention study by Amarenco P. et al. [7], patients after ischemic stroke or transient ischemic attack (with evidence of atherosclerosis) who had a target of LDL less than 1.8 mmol/L had lower cardiovascular risk than those with a higher LDL target (2.3 to 2.8 mmol/L). A meta-regression analysis (n = 222,149 patients in 23 trials), done by Shin J. and colleagues [8], demonstrated that each 1 mmol/L decrease in LDL cholesterol levels corresponded to a 23.5% stroke risk reduction.

Increase in LDL cholesterol is also progressively and significantly associated with a rise in the prevalence of carotid atherosclerosis (CA), even in the absence of other ‘traditional’ risk factors [9]. One of the potential mediators in this interplay between CA and LDL cholesterol is miR-33a—a microRNA which is up-regulated in CA [10] and positively correlated with LDL and total cholesterol levels in type 2 diabetes [11]. In our study we found that both strands of miR-33a (5p/3p) were associated with higher LDL cholesterol levels in CA patients in a univariable linear regression model.

miR-33a is an intronic microRNA, encoded within the *SREBP-2* gene, which encodes an important transcription factor actively involved in cholesterol metabolism [12]. Overexpression of miR-33a inhibits ABCA1/ABCG1 translation and cholesterol efflux, which is the key step to reverse cholesterol transportation in macrophages [13]. This has a major impact on atherosclerosis progression—most importantly, a number of studies have demonstrated that antagonism of miR-33 in vivo may increase circulating HDL and reverse cholesterol transport, enhancing the regression of atherosclerosis [14].

Using a cut-off value of 1.8 mmol/L for LDL we found that levels of miR-33a-5p/3p expression were significantly lower in CA patients who were below the target LDL levels. Interestingly, no correlation was found between miR-33a and HDL cholesterol levels—a link that is, perhaps, more pathogenetically appropriate. miR-33a-5p/3p expression was moderately correlated with degree of carotid stenosis (r = 0.44 and r = 0.38, respectively, *p* < 0.05), confirming its proatherogenic potential.

## 4. Materials and Methods

The study cohort and laboratory analyses were described in detail in Raskurazhev A. et al., 2022 [5]. Briefly, this was a prospective study that included 61 patients (median age 66.0 years, 55.7% male) with evidence of CA verified via ultrasound (included in analysis was the highest degree of stenosis in carotid arteries). Patients with malignancies, current infectious diseases, decompensated somatic pathology (including severe renal/hepatic disease), autoimmune disorders and stroke/myocardial infarction within 6 months were not included in this study. Previously, we described a differential expression of miR-33a-5p/3p in patients with advanced CA vs. control group [15].

Lipid profile (total cholesterol (mmol/L), triglycerides [TG] (mmol/L), HDL (mmol/L), LDL (mmol/L)) was measured on an automatic biochemical analyzer Konelab 30i (Thermo Scientific, Waltham, MA, USA) using Randox reagent kits (Randox Laboratories, Crumlin, UK).

Extraction of microRNA was performed using Leukocyte RNA Purification Kit (NORGEN Biotec Corp., Thorold, ON, Canada) according to modified manufacturer protocol. The polymerase chain reaction was performed starting with the reverse transcription step. Levels of miR-33a expression were normalized according to miR-16 levels—a stable endogenic reference control.

The following reagents and equipment have been used:

1. Validated 20X primers for hsa-miR: miR-33a-5p, miR-33a-3p (ThermoFischerScientific, Waltham, MA, USA)
2. Leukocyte RNA Purification Plus Kit (NORGEN Biotec Corp., Thorold, ON, Canada)
3. TaqMan™ Advanced miRNA cDNA Synthesis Kit (Applied Biosystems™, Thermo Fisher Scientific, Waltham, MA, USA)
4. Real-time CFX96 Touch amplifier (BioRaD, Hercules, CA, USA)

### Statistics

Statistical analysis was performed in R programming language (v. 4.1.0) using RStudio (version 1.4.1717) and the following downloadable packages: ‘tidyverse’, ‘reshape’, ‘corrplot’, ‘ggstatsplot’. Nonparametric tests were implemented. Discrete data are presented as frequency (%), continuous—as median (first quartile; third quartile). Comparison of two proportions was conducted with the Pearson z test with continuity correction. The Wilcoxon–Mann–Whitney test was used for two-sample comparisons. Relationship between variables of interest was analyzed using Spearman’s rank correlation coefficient (ρ). A univariate linear regression analysis was carried out with miR-33a expression level as independent variable. The 95% confidence interval was obtained using the formula: Estimate ± (Standard Error ×1.96). All statistical tests were two sided and results were deemed statistically significant if *p* value was < 0.05.

## 5. Conclusions

Overall, miR-33a up-regulation is associated with carotid atherosclerosis and may, in fact, be a key player by targeting cholesterol metabolism. A decrease in LDL cholesterol (<1.8 mmol/L) corresponded to lower levels of miR-33a, and both are, generally speaking, atheroprotective, yet the direction and causality of this association remains unclear. Anti-miR-33a therapy may be a viable option in targeting carotid atherosclerosis.

### Limitations

The study has several limitations: a small cohort size; this particular analysis was not pre-specified and was carried out post-hoc; the division into groups is arbitrary (though as per current lipid-lowering guidelines seems justified enough); the potential for selection bias (the patients were recruited only from one center); no long-term follow-up precludes establishing predictive conclusions; weak potential for direct causality assessment due to multiple possible confounders; absence of a control group comparable by gender and age without CA. This study represents an additional in-depth analysis of a previously described cohort of CA patients.

## Figures and Tables

**Table 1:** Main demographic characteristics of the study cohort.

	Study Population (n = 61)	LDL < 1.8 mmol/L (n = 26)	LDL ≥ 1.8 mmol/L (n = 35)	*p* *
Male, n (%)	34 (55.7)	12 (46.2)	22 (62.9)	0.30
Age, years (median [Q1; Q3])	66.0 [61.0; 71.0]	66.0 [62.0; 73.3]	65 [61.0; 70.0]	0.46
BMI, kg/m^2^ (median [Q1; Q3])	27.2 [25.5; 29.4]	26.4 [24.6; 27.6]	27.7 [26.3; 29.8]	**0.03**
Carotid stenosis degree, %	50.0 [35.0; 70.0]	50.0 [36.3; 70.0]	55.0 [37.5; 70.0]	0.60
Smokers, n (%)	21 (34.4)	9 (34.6)	12 (34.3)	>0.99
Stroke, n (%)	20 (32.8)	10 (38.5)	10 (28.6)	0.59
AH, n (%)	56 (91.8)	25 (96.2)	31 (88.6)	0.55
CHD, n (%)	24 (39.3)	11 (42.3)	13 (37.1)	0.89
MI, n (%)	10 (16.4)	8 (30.8)	2 (5.7)	**0.02**
DM, n (%)	26 (42.6)	14 (53.8)	12 (34.3)	0.21
AF, n (%)	7 (11.5)	6 (23.1)	1 (2.9)	**0.04**
ASA, n (%)	55 (90.2)	22 (84.6)	33 (94.3)	0.41
Anticoagulants, n (%)	13 (21.3)	7 (26.9)	6 (17.1)	0.54
Statins, n (%)	50 (82.0)	21 (80.8)	29 (82.9)	>0.99

* *p* indicates the *p*-value of the difference between columns 3 and 4. BMI—body-mass index; AH—arterial hypertension; CHD—coronary heart disease; MI—myocardial infarction; DM—diabetes mellitus; AF—atrial fibrillation; ASA—acetylsalicylic acid.

**Table 2:** Lipid profile of the study cohort.

	Study Population (n = 61)	LDL < 1.8 mmol/L (n = 26)	LDL ≥ 1.8 mmol/L (n = 35)	*p* *
LDL, mmol/L	2.0 [1.4; 2.7]	1.0 [0.96; 1.5]	2.6 [2.2; 2.9]	N/A
HDL, mmol/L	1.7 [1.3; 2.1]	1.5 [1.3; 2.2]	1.7 [1.5; 2.1]	0.31
Total cholesterol, mmol/L	4.8 [4.2; 6.0]	4.4 [4.1; 5.0]	5.2 [4.6; 6.9]	0.006
TG, mmol/L	1.4 [1.0; 2.0]	1.3 [0.9; 1.8]	1.8 [1.1;2.1]	0.06

* *p* indicates the *p*-value of the difference between columns 3 and 4; LDL—low-density lipoprotein, HDL—high-density lipoprotein, TG—triglycerides; N/A—not applicable.

**Table 3:** miR-33a expression levels by group.

	Study Population (n = 61)	LDL < 1.8 mmol/L (n = 26)	LDL ≥ 1.8 mmol/L (n = 35)	*p* *
miR-33a-5p, * 10^6^ copies	41.3 [36.8; 46.3]	37.4 [36.2; 45.4]	41.8 [38.7; 46.6]	**0.008**
miR-33a-3p, * 10^6^ copies	41.3 [36.9; 43.6]	38.3 [36.5; 40.8]	42.5 [40.9; 44.6]	**0.002**

* *p* indicates the *p*-value of the difference between columns 3 and 4.

**Table 4:** Univariable linear regression analysis (miR-33a-5p/3p levels—independent variables).

	miR-33a-5p	*p*	miR-33a-3p	*p*
LDL	0.03 [0.005–0.059]	**0.02**	0.03 [0.007–0.061]	**0.02**
HDL	0.006 [−0.010–0.022]	0.47	0.004 [−0.012–0.020]	0.60
Total cholesterol	0.010 [−0.040–0.060]	0.69	0.014 [−0.035–0.064]	0.58
TG	0.021 [−0.002–0.044]	0.08	0.021 [−0.002–0.043]	0.08

Shown are estimate coefficients [95% confidence intervals]. LDL—low-density lipoprotein, HDL—high-density lipoprotein, TG—triglycerides.

## Data Availability

The data presented in this study are available on request from the corresponding author.
